# Working conditions of intensive care unit nurses and quality of care during the pandemic: a qualitative study

**DOI:** 10.47626/1679-4435-2024-1277

**Published:** 2025-01-31

**Authors:** John Camilo Garcia, Yuliana Cárdenas, Isabela García, Susana Vallejo, Anibal Arteaga

**Affiliations:** 1 Nursing Care Research Group, School of Nursing, Universidad CES, Medellín, Antioquia, Colombia; 2 Family and Community Health Research Group, Corporación Universitaria Remington, Medellín, Antioquia, Colombia; 3 Masters’ degree in Quality in Health, Universidad CES, Medellín, Antioquia, Colombia

**Keywords:** nursing care, burnout, professional, working conditions, COVID-19, intensive care units, atención de enfermería, agotamiento profesional, condiciones de trabajo, COVID-19, unidades de cuidados intensivos

## Abstract

**Introduction:**

During the COVID-19 pandemic, an increase in patient demand was observed in intensive care units, as well as a deterioration of working conditions and quality of care.

**Objectives:**

To analyze the working conditions of the nursing staff of an intensive care unit in the city of Medellín during the COVID-19 pandemic and their relationship with the quality of nursing care.

**Methods:**

A qualitative study was developed in the adult intensive care unit of a third-level health care institution in the city of Medellin, Colombia. In-depth interviews were conducted with nine nursing professionals. The analysis was carried out in parallel with the collection of information throughout the research with the support of the qualitative data research software Atlas.ti.

**Results:**

The two central categories of this research were working conditions and quality of care. A dialogic relationship was observed between the two central categories of this research (working conditions and quality of care), in which good working conditions configured good quality of care. Similarly, as phenomena such as tiredness, fatigue, and work overload were observed, professionals described a subjective decrease in quality of care with a higher incidence of adverse events, absenteeism, and cost overruns.

**Conclusions:**

The deterioration of working conditions, particularly the work overload experienced by nurses during the COVID-19 pandemic, had direct consequences on their health and well-being, as well as on patient safety, quality of care, and health care institution costs.

## Abstract (es)

**Introducción:**

Durante la pandemia de COVID-19, se observó un aumento en la demanda de los pacientes en las unidades de cuidados intensivos, así como un deterioro en las condiciones laborales y la calidad de la atención.

**Objetivos:**

Analizar las condiciones laborales del equipo de enfermería en una unidad de cuidados intensivo de la ciudad de Medellín durante la pandemia de COVID-19 y su relación con la calidad de la atención de enfermería.

**Métodos:**

Estudio cualitativo realizado en la unidad de cuidados intensivos de adultos de una institución de salud de tercer nivel de la ciudad de Medellín, Colombia. Se realizaron entrevistas en profundidad a nueve profesionales de enfermería. El análisis se realizó en paralelo con la recopilación de información a lo largo de la investigación, con el apoyo del *software* de investigación de datos cualitativos Atlas.ti.

**Resultados:**

Se observó una relación dialógica entre las dos categorías centrales de esta investigación (condiciones de trabajo y calidad de la atención), en la cual las buenas condiciones de trabajo configuraron una buena calidad de la atención. Asimismo, al observarse fenómenos como cansancio, fatiga y sobrecarga de trabajo, los profesionales describieron una disminución subjetiva en la calidad de la atención, con mayor incidencia de eventos adversos, ausentismo y exceso de costos.

**Conclusiones:**

El deterioro de las condiciones laborales, especialmente la sobrecarga laboral experimentada por los enfermeros durante la pandemia de COVID-19, tuvo consecuencias directas en su salud y el bienestar, así como en la seguridad de los pacientes, la calidad de la atención y los gastos de las instituciones de salud.

## INTRODUCTION

Health care, as the scope of the nursing profession, is mediated by individual, institutional, social, and environmental factors.^1^ In this sense, the quality of nursing care is influenced by multiple human, work, technical, and academic factors, by social policies, and by hospital and health system structures.^2^ The nursing personnel is responsible for the quality of the care they provide; therefore, identifying cases of omission of care, as well the factors related to these omissions, makes it possible to take appropriate measures that involve restructuring services and improving nursing staff’s working conditions so as to contribute to resolve the problem of poor health care quality.^3^

Work does not consist only of struggling for subsistence and obtaining economic resources; it is rather a complex social fabric that allows people to recognize themselves as active and valuable members of a community, to develop one’s self esteem, and to promote social progress in general.^4^ Working conditions play a key role in the quality of nursing care, because they are related to all physical, social, and administrative factors that affect the work environment and, when not favorable, lead to job dissatisfaction and physical and psychological changes.^5^

Working conditions may have an influence on the quality of nursing care, with a higher incidence of adverse patient outcomes, longer hospital stay, greater morbidity and mortality, and a great economic burden in the provision of healthcare services.^6-8^ Furthermore, there is a vast literature on the psychological, emotional and social effects of work overload, outsourcing, multiple job holding, and low moral and economic recognition on the life of nursing professionals.^9-12^

Due to the few employment guarantees offered in South America, a great number of workers have moved to other regions in the world due to conditions of economic development, searching for job opportunities and working conditions that in turn allow them to attain better living conditions, with better payment conditions and lower workload, in which the nursing professional is dignified and valued.^13^ There are great differences between low, medium and high income countries in terms of recognition of the nursing profession: in the United States and Canada it receives a wide professional recognition and the development of advanced practice in nursing and clinical specialties,^14^ whereas in countries of South America and the Caribbean professional recognition is incipient,^15^ a geopolitical scenario that affects working conditions and professional development.

Nursing professionals’ quality of life has been increasingly affected and undermined by both physical and mental exhaustion from excessive workload and by poor working conditions in health institutions, with the risk of experiencing fatigue and exhaustion due to occupational stress caused by this situation day after day. Most studies on burnout^16^ conducted with nursing professionals agree that this situation also implies metabolic changes, especially in night-shift workers, as well as stress-related, mechanical and postural diseases resulting from work overload faced by nursing professionals today, which are framed within a larger set of psychological and emotional conditions.^17,18^

These medical conditions affecting health care workers were exacerbated in the context of the COVID-19 pandemic, since there was a significant deterioration in working conditions, especially in intensive and emergency care areas, since they are the most prone to turmoil, stress, rush, moral suffering, and decision-making in tense environments about complex processes such as health, disease, life, and death.^19,20^ The pandemic had such an impact on health care professionals’ working conditions that que the term “second victims” of the pandemic has been often used.^21,22^

In this sense, the aim of the present study was to analyze the working conditions of the nurse staff working in an intensive care unit (ICU) in Medellín, Colombia, during the COVID-19 pandemic and their relationship with quality of nursing care.

## METHODS

This qualitative study was conducted within an interpretative paradigm, which seeks to understand the world of subjective experience and everyday life from the point of view of individuals who experience it.

### POPULATION AND SAMPLE

This research was carried out at an adult ICU of a third-level healthcare institution (HCI) in Medellín, Colombia. The study population consisted of nursing professionals working at the COVID-19 ICU during 2020 and 2021 and who voluntarily accepted to participate in the study. Participants were selected using an intentional snowball sampling strategy. A member of the research team (SV), who was affiliated with the institution, was responsible for connecting and establishing contact with study participants. Varied profiles of nursing professionals were selected, in order to contribute to diversity, so that they could represent different realities and provide complementary perspectives in their statements.

### CRITERIA FOR PARTICIPATION AND POSSIBLE CAUSES OF INFORMATION LOSS

The criteria were considered in the selection of the sample: a) nurses with at least 2 years of experience in the ICU and b) nurses that had worked in the ICU during 2020 and 2021.

Possible exclusion criteria were defined as nursing professionals with preexisting mental disorders. However, no participants were excluded according to the established criteria.

### INFORMATION COLLECTION

Information was collected by two nurses (SV and YC), both with experience in intensive care and high dependency units, supervised by JCG, with experience in ICU and qualitative methods. This specific experience in intensive care and high dependency units and experience in ICU and qualitative methods takes into account a collaborative approach and appropriate supervision during the data collection process, which strengthens the validity and reliability of the obtained findings. Before field immersion, narrative descriptions of the experiences of researchers themselves were conducted, with the framework of the phenomenological concept named *epojé.*

The participating individuals were approached individually; location and data of the interview were arranged with those who manifested their interest in participating in the study. Overall, 15 professionals were approached, of which nine composed the final sample, selected based on participants’ compliance with inclusion criteria and availability. Collection of basic demographic data included information about age, gender, educational level, and years of work experience. Age was divided into two categories: from 28 to 34 years (four participants) and from 35 to 45 years (five participants); moreover, all participants were women. Educational level was classified into three groups: undergraduate (five participants), post-graduate (three participants) and master’s degree (one participant). Work experience was divided into two groups: 3 to 10 years (seven participants) and more than 10 years (two participants).

Interviews were conducted by the research team, after standardization, awareness, and initial field immersion, and information was gathered in the 1st semester of 2023. All interviews took place in person, maintaining physical distancing and using personal protective equipment. With participants’ permission, sessions were audio-recorded in order to capture all elements of interactions during the interviews, which lasted from 22 to 44 minutes. Subsequently, investigators transcribed the interviews verbatim using Microsoft Word.

### DATA ANALYSIS

The data analysis process was conducted iteratively and in parallel with information collection throughout the entire research, using the qualitative data analysis software Atlas.ti with license R-D4B-0BA-DC4-01E-7BE-D63 and license ID L-DD1-9E5. Analytical tools based on the grounded theory were applied, starting with a preliminary reading of the interviews to identify units of meaning. An exploratory analysis was performed through counting and creation of word clouds, from which prepositions were removed. Subsequently, open codification was conducted using substantive and in vivo codes, maintaining a constant comparative analysis by grouping and ungrouping data. As interviews progressed and constant comparative analysis was performed, it was possible to advance towards a deeper understanding of the theme, with led to the second stage of analysis: axial codification. In this stage, data were reorganized, establishing connections between the categories of healthcare quality and working conditions, as well as between the subcategories that emerged during the analysis^23^ to determine the semantic categories according to the density and rooting of data, evaluated using co-occurrence analysis and semantic networks.

Once network graphs were built, statements were associated with each of the codes for the interpretation and analysis of themes and subthemes. The codification process was conducted independently by each member of the research team. To avoid redundant codes, the project versions were exported and imported; furthermore, codes and their relationships were discussed to establish a consensus. During the data collection and analysis process, the research team adopted an attitude of constant reflexivity; this is the phenomenological *epojé* required to allow oneself to be surprised by the phenomenon of interest without conditioning researchers’ perspective by clinical experience in the ICU during the pandemic, i.e., to counteract their own perspective as one of the methodological rigor criteria.

This study was approved by the Research Committee of Universidad CES through protocol no. 211, project code 1102 of 2023, and by the HCI’s committee through protocol no. 204 of 2023. The researchers declare no conflicts of interest.

## RESULTS

A dialogical relationship was observed between the two key categories of this research (working conditions and quality of care), with good working relationships being related to good quality of care. Similarly, as phenomena such as tiredness, fatigue, and work overload were observed, professionals described a subjective reduction of quality of care, with a higher incidence of adverse events, absenteeism, and costs overruns. These poor working conditions and low quality of care in the narratives if participants in this study were associated with guilt, resentment, and long-term distress. Next we present a description and analysis of categorical and subcategorical cores.

### WORKING CONDITIONS

*“We work more and more and achieve few results and less recognition”* (EE8).

Some statements revealed work overload and feeling of frustration from the professionals participating in the study: *“I felt that we work more, but we saw fewer results. It was frustrating, because since one arrived to work, one kept doing and doing, sometimes one even had to work a little longer because one could not keep up with everything one had to do” (*E7). This led to extended working hours and an increase in the complexity of each shift, as reported in the following statements: *“Workload increased 1000%, I would say, definitely many people knew that shifts would be like they used to; indeed, we’ve never had shifts like those at the unit before”* (E4); *“There were days when we left work at 10 p.m., 9.30 p.m., 10.30 h a.m. in a post-shift, and this was because we tried to give our best”* (E5). The foregoing reveals how working conditions affected the quality of health care provided to patients during the pandemic.

Since intensive care units were overcrowded, the HCI under study was confronted with the need to increase the number of ICU beds in an extension room, which did not have comfortable working conditions for the personnel, as evidenced by the following statement:

“*I wasn’t prepared at the beginning, it was hell, I can’t never compare the heat I felt in this room with going to San Andrés or Cancún, (…). Never! It was a sauna, because all the time you were wearing gown, gloves, elastomeric pump, googles, face shield, and there was the patient here, it means, 50 cm from you… Intubated, in the prone position, and with other two assistants, it was like we made rotations to be able to leave, breathe air, and pee (urinate). And sometimes we felt alone, is sort of a no-man’s land, sometimes not even the doctor showed up... we ran out of things, we were there, praying for someone to throw us some gauze, so to speak, we were thrown”* (E4).

The working conditions described by the participants were related to occupational health and to physical, mental and social wellbeing. Work overload was a constant complaint and the main mechanism for occupational diseases, physical exhaustion, absenteeism, demotivation, resignations, and burnout. “*Work overload was the common denominator, we knew that work would be heavy, but expectations were fully exceeded by the cruel ruthless reality”* (E9). *“There were shifts when one started and was literally not able to sit down even for a second, my smartwatch counted more than 20,000 steps, my feet hurt, and on the other day I woke up still with a burning sensation on the soles of my feet and didn’t want to go to work”* (E8). As the pandemic advanced, working conditions became increasingly more difficult, leading to workers’ resignations, occupational disabilities, and feeling of burnout in the part of the personnel who were not sick yet, which generated additional overload and promoted a vicious circle consisting of work overload, occupational disease, absenteeism, and greater overload. This is described in the following statements: “*Sometimes one waited until 10 a.m. for someone to replace colleagues who did not come to work because they took a sick leave disabled or workers who resigned and had not been replaced yet, so one left work more tired* (E7). *“I didn’t like answering the phone or checking text messaging groups, there was always someone looking for a colleague to replace those who got sick with COVID or with low back pain or who simply took a sick leave to rest”* (E8).

The tired and fatigated staff of this HCI started to perceive changes in the meaning of work itself and to lose interest in caring for patients. These feelings se intertwined with feelings of helplessness and guilt that persist until today and is still affecting health care professionals:

*“There were days when one arrived work not willing to talk, not willing to work, when one was only counting down hours and minutes to leave this confinement with a gown and a mask, and so much pain, sometimes I didn’t want to go to work, but I feel bed, and still feel bad today, because I feel that I didn’t do things the best way possible (crying), although I try to comfort myself thinking that, given what was happening, we did what we could”.*

The context of the pandemic led to shortage of economic, human and technical resources. However, the professionals emphasize the institutional effort for managing all the resources. *“Despite all difficulties, our payment was never missed, and we never ran out of masks, because when they were about to run out, we made fundraising efforts among everybody”* (E4). *“We saw that one knocked on the door of other institutions, NGO and government to obtain human and financial resources, it was always heard that someone made a donation or became a volunteer, and this helped”.*

However, despite this institutional effort, in the everyday life inside the ICU, healthcare professionals experienced feelings of abandonment with respect to decision making, to teamwork, and to how to solve shortage of personnel and patient overload. This facilitated errors, healthcare failures, and dehumanization. “*With shortage of medications and of personnel and with so much work, of course one made mistakes, from the slightest one (good relationship skills), up to complex situations such as installing ECMO, and of course it had consequences for patients and for everyone”* (E1).

There were also social and political determinants that shaped two perspectives in professionals’ imagination (heroes and villains), and a certain social stigma favored a feeling of exhaustion and loss of interest in the subject of health care. “*We went from being applauded in the streets and receiving free housing to being systematically excluded from public spaces”* (E2), *“I was often verbally attacked, I was told that I could not come in, they literally feel disgust at me”* (E3).

*“One thing that discouraged me was listening people and even the news saying that they are killing patients at the ICUs, that they are intubated to gain money, I’d rather saying nothing at the beginning, but then I decided to change my mind and ended up in long and heated discussions, it was unfair that one was killing themselves there and people were saying that”* (E4).

**Figure 1:**
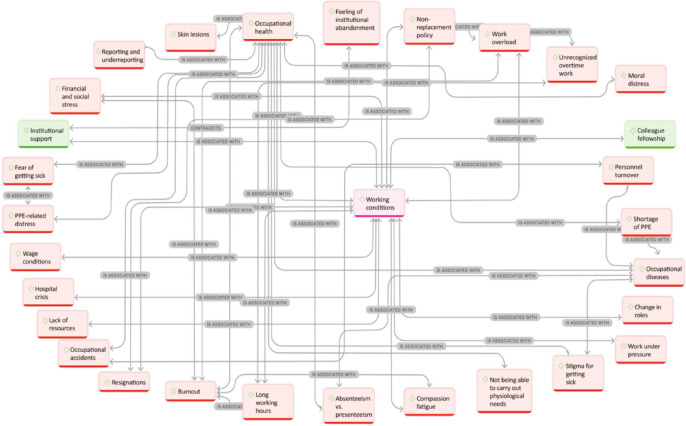
Categorial map of working conditions.

The categorical map corresponding to working conditions during the pandemic is presented in Figure 1. At the center of the figure there is one of the main categories of this study (working conditions), which appears in pink because all study participants were female nurses. A greater convergence of connections indicates higher density and rooting of codes throughout participants’ statements, as in the case of health conditions at work, work overload, and burnout. The complex relationships and connections allow for understanding how working conditions are multidetermined and determine a wide variety of situations at the same time. In generic terms, positive aspects related to working conditions were highlighted in green, especially colleague fellowship or interprofessional collaboration and teamwork were a positive aspect; however, the other relational dimension, that of workers’ interpersonal relationships, was considerably affected. Institutional support was described as a positive aspect, although it was an issue of disagreement among participants, especially to deficiencies in economic and structural resources. The other aspects that, in participants’ opinion, influenced or were influenced in a negative manner by working conditions during the pandemic are highlighted in red.

### QUALITY OF CARE AS A TIME-TRANSCENDING AND SOCIAL PHENOMENON

**Figure 2:**
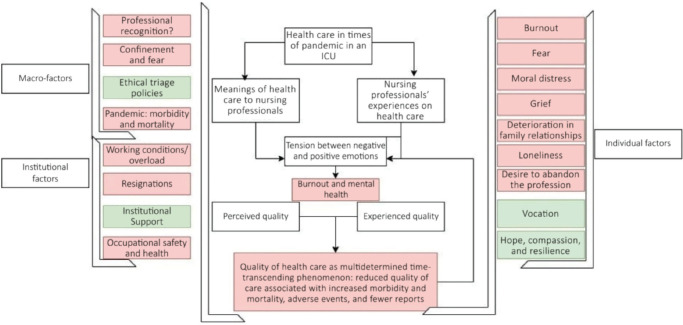
Quality of care as a time-transcending phenomenon influenced by individual, institutional, and social factors. Own creation using Draw.io. ICU = intensive care unit.

Quality of nursing care as described by ICU participants was influenced by macro- (social and governmental), meso- (institutional) and micro-factors (individual and patient), as presented in Figure 2, which represents the categorical network of working conditions among ICU nursing personnel during the pandemic.

With regard to the experiences related to the quality of health care component, a tension was observed between positive and negative emotions and experiences: some professionals felt apprehension, fear, suffering, and burnout, whereas others believed that the pandemic was an opportunity to make nursing healthcare visible in critical contexts, allowed for the establishment of new public policies, and to consider professional vocation as a crucial factor crucial in professional development. In this sense, one of the participants stated the following: “*I felt that I couldn’t take it anymore, with each night I spent seeing people die and listening criticisms was like I died little by little”* (E9), whereas another professional considered that:

“*I know that it was hard, but the pandemic allowed us to grow as a country, as an institution and as a person, national protocols were established, new policies, great vaccination coverage, and an at least symbolic recognition of healthcare professionals’ work. Additionally, in the midst of so much pain, of so many hard times, here we are, holding on and moving ahead, I don’t know if it’s because of resilience, vocation, or simply love, but I believe that it’s something to highlight, that today many of us still work at the ICU and we are increasingly more committed to what we do and (long silence) and maybe preparing themselves to future pandemics?”* (E8).

However, work overload, physical and mental fatigue, loss of interest in work, and low recognition were factors that influence the detriment in care quality. There was an increase in adverse events during the pandemic, followed by a reduction in their reporting. A study participant said: *“I believe that there was an increase, but it was underreported, it means, because of fear, people neglected some things. They were not reported or were hidden, thinking that the person would die anyway”* (E4).

Errors in prescription, treatment, programming of pumps, and in administration of sedative, vasoactive, inotropic, and lipid-lowering drugs were identified. Several statements reflect this situation, such as the following: *“I witnessed numerous errors in the administration of medicines; the presence of underreporting was impressive”* (E6).

*“The pumps beeped continuously. You were inside changing a patient and, outside, norepinephrine pump was beeping. You didn’t have anybody to help you with that; there was a constant beeping showing that the drug was running out and, inside the unit, there was nobody to restart or continue the infusion”* (E4).

This used to generate feelings of guilt, pain, and moral distress:

*“One felt that one was doing things badly, I felt helpless every time a patient died, sometimes I got home to simply lock myself and cry. Sometimes these memories come into my mind, when I had to take bags to wrap patients and unintentionally start crying again”* (E1).

“*One wished that it never happened again, I had a terrible cystitis because I could not use the bathroom, I was depressive and incapacitated for work for a long time, and when I went back I got COVID, but I felt bad because adversely affected my colleagues, and I was worried that they felt I was abandoning them at the worst possible time”* (E3).

## DISCUSSION

The crisis derived from the COVID-19 pandemic was an unprecedented event that had a profound impact on all contexts in which people perform, encompassing both social and family spheres and the work-related sphere.^24^ Health institutions found themselves immersed in a crisis scenario marked by the national and international repercussions on the social and health domains. This resulted from changes both in health care practice and routines, as well as in the way professionals establish connections with patients, relatives and other members of the health care team. These changes were reflected in the reports and subjective experiences of the participants involved in this study.^25,26^

Distress, suffering, posttraumatic stress, and burnout have been the sequel of the pandemic in health care professionals.^22^ Although the hospital crisis derived from COVID-19 seem to be over, the professionals assessed in this remember those difficult times with pain, some of them still cry easily and have feelings marked by guilt; therefore, there is a need to promote spaces for dialogue and externalization of feelings. Although the aim of this study was not to provide nor to evaluate psychological support, these spaces were facilitated through dialogic cathartic processes and improved in the health care institution to favor psychological support. The foregoing has also been described by other authors.^27-29^

Nurses’ working conditions remain a major concern. After the pandemic, laws, agreements, and work groups have been implemented in countries such as Colombia to favor a dialogical space that advocate for fair and decent working conditions for all nurses. The World Health Organization has promoted some strategies such as Nursing Now and “The Year of the Nurse and Midwife”. However, these spaces seem to be insufficient in the geopolitical and social context in which nursing practice is not valued.^11^

Recognition of nursing profession and work represent the minimum requirement, considering the essential importance of this profession both for provision of healthcare services and for society at large. Lack of recognition and visibility of nurses’ role, especially in environments such as ICU and other health care services, has been related to professional burnout and emotional exhaution.^10^ The reports collected through the interviews describe how experiences related to diseases, suffering, and death, exacerbated by work overload, shortage of personal protective equipment, and poor human relationships, contribute to greater burnout and emotional fatigue. These findings coincide with the results of previous studies.^11,12^

This qualitative study contributes to the understanding of nursing personnel’s experiences and perceptions about working conditions in such a challenging context as the pandemic. In the context of qualitative studies, it is important to acknowledge their limitations in generalization of findings; however, the qualitative approach provides a valuable perspective to inform policies and practices that improve working conditions and emotional well-being of the nursing personnel in situations of crisis. The findings of this study provide specific recommendations to improve support and care to ICU nursing personnel for the future, in which healthcare professionals’ mental health is still a crucial issue in the context of occupational medicine and occupational health.

## CONCLUSIONS

Deterioration of working conditions, especially work overload experienced by the nursing personnel during the COVID-19 pandemic, had direct consequences on their health and well-being, as well as in patient safety, quality of care, and health institution costs. Throughout the COVID-19 pandemic, high demand for health services evidenced the reduction in the availability of resources, both human and material. These difficulties directly affected the quality of care provided to patients and contributed to the perception of excessive workload by the nursing personnel and to feelings of guilt, moral distress, and sadness that still persist 2 years after the pandemic.
